# Local Insect Damage Reduces Fluctuating Asymmetry in Next-year’s Leaves of Downy Birch

**DOI:** 10.3390/insects9020056

**Published:** 2018-05-11

**Authors:** Mikhail V. Kozlov, Dmitry E. Gavrikov, Vitali Zverev, Elena L. Zvereva

**Affiliations:** 1 Department of Biology, University of Turku, 20014 Turku, Finland; vitzve@utu.fi (V.Z.); elezve@utu.fi (E.L.Z.); 2 Pedagogical Institute of Irkutsk State University, Nizhnyaya Naberezhnaya 6, 664011 Irkutsk, Russia; dega.irk@gmail.com

**Keywords:** background insect herbivory, *Betula pubescens*, delayed response, fluctuating asymmetry, leafminers, leafrollers, within-plant heterogeneity

## Abstract

Insect herbivory imposes stress on host plants. This stress may cause an increase in leaf fluctuating asymmetry (FA), which is defined as the magnitude of the random deviations from a symmetrical leaf shape. We tested the hypothesis that differences in leaf FA among individual shoots of downy birch, *Betula pubescens*, are at least partly explained by local damage caused by insects in the previous year. Unexpectedly, we found that in the year following the damage imposed by miners, leafrollers and defoliators, damaged birch shoots produced leaves with lower FAs compared to shoots from the same tree that had not been damaged by insects. This effect was consistent among the different groups of insects investigated, but intra-species comparisons showed that statistical significance was reached only in shoots that had been damaged by the birch leaf roller, *Deporaus betulae*. The detected decrease in leaf FA in the year following the damage agrees with the increases in shoot performance and in antiherbivore defence. The present results indicate that within-plant variation in leaf FA may have its origin in previous-year damage by insects, and that FA may influence the current-year’s distribution of herbivory.

## 1. Introduction

Trees are modular organisms consisting of repetitive multicellular semi-independent units, such as leaves, shoots, branches, stems and roots. The theoretical and practical importance of studying the effects of herbivory on individual modules has long been appreciated [1]; however, little is known regarding the consequences of localised herbivory on the resulting performance of both the affected module and the whole plant.

Previous studies demonstrated that local damage experienced by one leaf or several leaves of the same shoot may cause shoot-specific responses in the year following the damage, and that these responses can contribute to intra-plant heterogeneity in leaf quality for insect herbivores [2,3]. In particular, insect damage inflicted on leaves of an individual shoot can decrease the probability of herbivore attack on the next year’s foliage produced by the same shoot, compared with attacks on shoots of the same tree that had not suffered insect damage in the previous year. In other words, the following year’s leaves, from shoots that previously experienced herbivore damage, exhibit a higher level of antiherbivore defences [3]. However, the cues used by plant-feeding insects to avoid these shoots that have been damaged in the previous year remain to be established.

Leaves on an individual plant can differ greatly in many traits [4], including leaf fluctuating asymmetry (FA), which is defined as the magnitude of the random deviations in symmetry of the leaf shape. Many researchers have explored correlations between average levels of leaf FA (based on 10–20 leaves per plant) and different indices of insect herbivory. These observational studies did not reach an agreed conclusion; some of them reported higher herbivory on plants with higher FA [5,6,7], while others discovered the opposite pattern [8], or reported the absence of any correlation between plant FA and herbivory [9,10,11].

In contrast to among-plant variation, only a handful of studies explored the link between the FA of individual leaves of the same plant individual and their preference by insects and/or performance of insects fed with these leaves. Specialist leafmining and defoliating insects generally distinguish between nearly symmetric and highly asymmetric leaves of the same plant [12,13], but surprisingly, do not distinguish between identically sized disks cut from these leaves [13]. Many insects evaluate leaf shape when selecting feeding or oviposition sites [14,15], raising the possibility that leaf asymmetry serves as a cue in the selection of individual leaves by herbivores within a plant crown. This selection could optimise a herbivore’s feeding strategy, because insect preference for either low-FA or high-FA leaves matches the insect performance when fed with these leaves [13].

In several study systems, plants that undergo severe defoliation due to insects demonstrate an increase in leaf FA in the year following the damage [8,16,17]; this effect is directly proportional to the degree of the previous year’s herbivory, at the scale of individual trees [18]. At the same time, we are aware of only two studies that explored the consequences of localised damage on subsequent leaf FA at the scale of individual shoots [18,19]; both reported a general lack of any effect of herbivore feeding on leaf size or FA in the subsequent year. The shortage of information on the shoot-specific effects of herbivory on subsequent leaf asymmetry prompted the present study. Here, we have tested the hypothesis that shoots damaged by insects produce leaves with higher FA in the year following the damage than is observed in leaves of neighbouring shoots that were not damaged in the previous year.

## 2. Materials and Methods

### 2.1. Study System

Downy birch, *Betula pubescens*, is widely distributed in Eurasia and forms stable climax forests at the northern edge of its range. In contrast, in more southern regions, birches are colonists in natural and human-induced secondary successions. Birch leaves are damaged by several hundreds of insect species [20], but we restricted our study to the effects caused by the following insects: (1) *Deporaus betulae* (Coleoptera: Rhynchitidae); (2) *Eriocrania semipurpurella* (sensu lato) and *E. sangii* (Lepidoptera: Eriocraniidae); (3) *Stigmella* spp. (Lepidoptera: Nepticulidae); (4) *Parornix* spp. (Lepidoptera: Gracillariidae); (5) leafrolling/leaf-tying larvae—several species of small moths building nests from birch leaves (Lepidoptera: Tortricidae and Gelechiidae); and 6) several species of mining sawflies from the genera *Fenusa*, *Profenusa*, *Fenusella* and *Scolioneura* (Hymenoptera: Tenthredinidae). For characteristics of damage imposed by these herbivores consult [3].

### 2.2. Data Collection

The study was conducted in two localities in southwestern Finland, near Turku (Jäkärlä: 60°32′ N, 22°33′ E; Raisio: 60°29′ N, 22°09′ E). Turku is surrounded by boreo-nemoral forests, and the mean July temperature is +17.5° C. The study sites (ca. 50 × 200 m each) were chosen in sparse mixed forests with abundant young (up to 3 m tall) birch trees. During the study years (2008–2009), the proportion of birch foliar biomass removed by insects by the end of the growth season at our study sites was about 10%, and the densities of all herbivorous insects were at their background levels.

We tagged current-year vegetative shoots of downy birch damaged by the selected groups of herbivores (experimental shoots hereafter) on 18–23 August 2008. The shoots included in our study had to fit the following criteria: (1) the leaf mine or roll/nest produced by a target herbivore was of its final size (i.e., we excluded uncompleted mines and nests where a herbivore died at an early developmental stage); (2) foliar losses due to damage of the selected shoot by nontarget herbivores were less than 1%; and (3) a control (undamaged or nearly undamaged) shoot of the same type was available on the same tree, and it resembled the corresponding experimental shoot in size, leaf number, and position within the crown. Data collection in 2008 was non-destructive: we measured the length of each leaf in selected experimental and control shoots, and visually estimated the percentage of leaf area injured by herbivores.

The year after the damage occurred (2009), we collected all the shoots that developed from buds of the tagged shoots after the termination of shoot growth (21–23 July). Leaf size and biomass were measured as described in [3]. At that time, we had not planned to conduct measurements of leaf FA, but the leaves were preserved in small envelopes, and the larger part of the leaves remained flat and unbroken after drying and weighing.

In 2017, M.V.K. selected 1–2 of the best preserved leaves from each of 50 experimental shoots and from 50 paired control shoots and coded these leaves (184 in total) in a blinded fashion that prevented the determination of the leaf origin from the leaf code. This procedure assured that leaf measurements were not prone to confirmation bias [21]. The leaves were mounted on strong paper and scanned at 600 dpi resolution. The width of the left and right halves at the midpoint between the base and the apex of leaf lamina was measured from images of these leaves by D.E.G., following a protocol described in an earlier study [22]. In brief, the middle of the midrib was identified using a distance tool in Image J 1.49 V, and the perpendicularity of measurement line to the midrib was achieved by placing a new layer with an image of a cross over the measured image. The second measurement was performed two weeks after the first, and blinded to the results of the first measurement.

### 2.3. Data Analysis

We first conducted a mixed-model ANOVA to detect any evidence for directional asymmetry and FA relative to measurement error [23,24]. In this analysis, the leaf side was considered a fixed factor, and the individual leaf a random factor (SAS MIXED procedure [25]). We evaluated the reproducibility of measurements by calculating the index ME5 = [MS_i_ − MS_m_]/[MS_i_ + MS_m_], where MS_i_ and MS_m_ are the interaction and error mean squares from a sides × individuals ANOVA [23]. This index expresses FA variation as a proportion of the total variation between leaf sides, which includes the variation due to both FA and the measurement error calculated from two independent measurements.

The size-corrected FA values were calculated from the width of the left and right leaf halves (WL and WR, respectively) as follows: FA = 2 × abs(WL − WR)/(WL + WR). This index was widely applied in earlier studies of plant leaf FA [26,27,28,29], and its use is justified by the significant positive correlation between the absolute difference in the measured character between the left and right leaf halves and leaf size, whereas size-corrected FA values were independent of leaf size (data not shown).

The FA values were square-root transformed to meet a normality assumption, averaged between two subsequent measurements of the same leaf, and then between two leaves collected from the same shoot. The shoot-specific values of foliar damage measured in the year preceding leaf sampling were log(1 + √x) transformed prior to analysis. Both FA and foliar damage were analysed with a linear mixed model (SAS GLIMMIX procedure). We considered treatment (experimental vs. control shoots), herbivore identity (six groups, see above), and their interaction as fixed effects, whereas the study site and the sampled tree (nested within a site) were treated as random effects. We facilitated accurate *F* tests of the fixed effects by adjusting the standard errors and denominator degrees of freedom using the latest version of the method described in [30]. This analysis showed that insect identity did not explain the variation in leaf FA; therefore, at the final stage, we combined shoots damaged by all groups of insects and compared untransformed FA values between paired experimental and control shoots by the conservative non-parametric Wilcoxon signed-rank test (SAS UNIVARIATE procedure [25]).

## 3. Results

### 3.1. Identification of Fluctuating Asymmetry

Analysis of our data demonstrated a highly significant side × leaf interaction (*F*_183,368_ = 398.8, *p* < 0.0001), confirming the existence of FA in our sample and our ability to identify FA using repeated measurements of the given accuracy. The left half of the analysed leaves was, on average, 1.8% wider than the right half (*F*_1,183_ = 6.13, *p* = 0.01), whereas the average absolute difference in width between leaf halves was much greater (8.5% of leaf half-width). Variance components from the mixed model ANOVA indicated that measurement error (ME5) accounted for 0.5% of the variation in FA.

### 3.2. Effects of Previous-year Herbivory on Fluctuating Asymmetry

**Figure 1:**
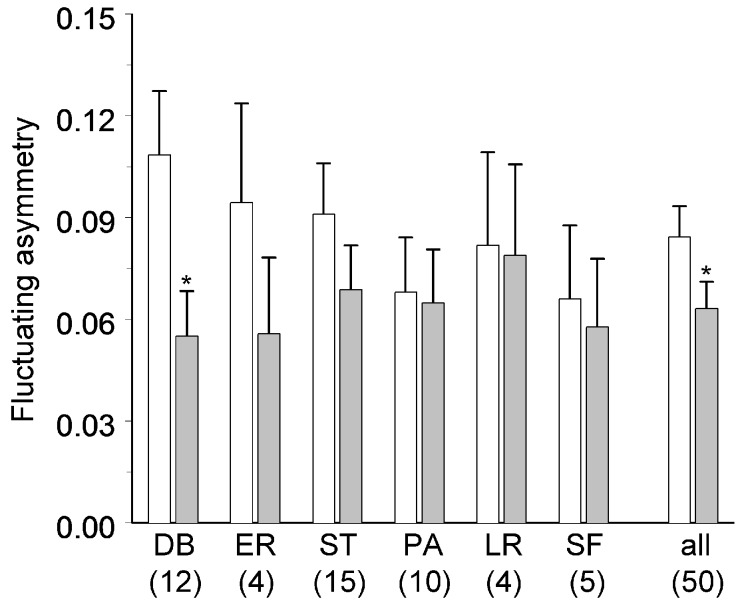
Fluctuating asymmetry of experimental (grey) and control shoots (white) of *Betula pubescens* in 2009, the year after the experimental shoots were damaged by the following insect herbivores: DB—*Deporaus betulae*, ER—*Eriocrania semipurpurella* and *E. sangii*, ST—*Stigmella* spp., PA—*Parornix* spp., LR—leafrolling larvae, SF—mining sawflies, all—all groups pooled. Values are estimated marginal means; bars indicate standard errors; sample sizes (numbers of shoot pairs) are shown in parentheses. An asterisk indicates significant (*p* < 0.05) difference between experimental and control shoots (individual herbivores: *t*-test; all herbivores combined: Wilcoxon signed-rank test).

The previous year’s losses of foliage to insects (Table S1) were significantly higher in the experimental shoots than in the control shoots (Table 1; mean ± S.E.: 16.3 ± 1.9% and 2.0 ± 0.4%, respectively). Mixed model ANOVA demonstrated that the FA (Table S1) tended to be lower in leaves collected from the experimental shoots than from the control shoots (Table 1). This difference in FA between the experimental and control shoots did not depend on the identity of the insects that had inflicted damage in the previous year (Figure 1, Table 1), but reached statistical significance only for the damage caused by the birch leaf roller, *D. betulae* (Figure 1). The paired Wilcoxon test confirmed that leaf FA was significantly lower in experimental shoots (all herbivores combined) than in control shoots (*S* = 271.5, *p* = 0.0074).

## 4. Discussion

Our study is the first to demonstrate that local and relatively minor damage (on average, a 16% loss of foliar biomass to insects) may change the symmetry of leaves produced by the affected shoots in the year following the damage. This result was predictable, because herbivory was previously reported to increase leaf FA at the plant-specific and site-specific scales [8,16,17]. However, the direction of the effect observed here, namely, the *decrease* in FA as a response to herbivory, was unexpected. Typically, FA either increases or remains unchanged with an increase in environmental stress [23,31,32], whereas its decrease is usually observed following alleviation of stress [33,34]. Assuming that a larger FA reflects greater developmental instability resulting from environmental or genetic stress [23,35], our result suggests that the minor damage inflicted to an individual shoot by insect herbivory caused a partial alleviation of the stress routinely experienced by plant shoots, and/or it imposed an increased stress on nearby undamaged shoots on the same plants.

We recognise that our study design poses some limitations on the interpretation of the results. Indeed, the distribution of damage between shoots was not random, but resulted from shoot selection by ovipositing females. However, we are not aware of any data showing that insects can recognise and preferentially infest shoots that have the potential to produce leaves with low FA during the year following oviposition. Furthermore, we did not plan measurements of leaf FA when we established this experiment, so our selection of experimental or control shoots was not affected by leaf asymmetry. Therefore, we believe that our result was not influenced by a non-random selection of experimental or control shoots.

The damage imposed by a community of plant-feeding insects when at their ‘normal’ low population densities is termed background insect herbivory [36]. The direct losses of foliar biomass due to background herbivory are relatively minor, so these losses are often considered negligible (e.g., [37]). However, steadily accumulating data now reveal that minor damage can have major effects when acting continuously, due to activation of plant defences, triggering of premature leaf abscission, and disturbance of plant growth and reproduction (reviewed in [38]). A recurring suggestion is that the mechanisms underlying plant resistance and tolerance to episodic severe damage versus minor chronic damage due to insects are likely to differ [3,39,40]. Our results corroborate this hypothesis, as we found that the leaf FA decreases in the affected shoots in the year following minor local damage, in contrast to a reported [8,16,17] increase in leaf FA in a year following severe defoliation of the entire plant.

Our finding of a decrease in leaf FA in the year after shoot damage by insects looks less anomalous when compared with data on shoot performance. In the year following the damage, the damaged shoots of downy birch (the same shoots from which we measured FA in the current study) produced 13.8% more biomass than that of undamaged shoots from the same tree [3]. This pattern, as well as the decrease in leaf FA, were most pronounced in shoots damaged by birch leaf roller, *D. betulae*, which inflicts the most extensive damage upon birch leaves of all our study species [3]. This compensatory growth was previously interpreted as a consequence of an increase in primary metabolism in the affected shoot and/or in translocation of assimilates to the site of damage, and these effects were suggested to occur due to hormonal mechanisms triggered by insect damage [3]. This redistribution of resources may be a proximate mechanism that could explain the observed differences in leaf FA between shoots that were and were not damaged by insects during the previous year. Thus, substantial variation in leaf FA observed among individual leaves of a plant [41] at least partly reflects the previous-year distribution of damage inflicted by insects within the plant crown. However, the ecological and/or evolutionary significance of this variation remains to be established.

A previous (yet unpublished) study demonstrated that specialist defoliators distinguish between nearly symmetric and highly asymmetric leaves of the same plant [13]. Thus, leaf asymmetry could conceivably serve as a cue in the selection of individual leaves by herbivorous insects within a plant crown. Controlled experiments demonstrate that insects prefer high-FA leaves to low-FA leaves of downy birch [13], and that insects show higher growth rates when consuming leaves from high-FA than from low-FA trees [42]. Our current finding of a lower FA of leaves produced by previously damaged birch shoots is in line with these results, because the leaves of these shoots which grew in the following year were less frequently damaged by insects than were the leaves of shoots that had experienced little or no previous-year damage [3].

Studies exploring the impacts of insect herbivory on plants usually control for plant-specific losses of leaf biomass, while commonly ignoring the distribution of damage within a plant. At the same time, a specific spatial pattern of damage may be more detrimental to plant fitness than other patterns [4]. Therefore, selection of leaves based on their asymmetry may not only increase the fitness of insect herbivores, especially those that cannot change their feeding sites in the course of larval development (like miners and gallers), but may also decrease the susceptibility of the whole plant to background insect herbivory. In particular, some observations suggest that evenly distributed herbivory results in a smaller decrease in growth and reproduction of the whole plant, when compared to the same level of herbivory concentrated on only one part of the crown [43]. We now extend this hypothesis to between-year differences in the distribution of damage, and we suggest that insect preference for shoots that have not been damaged in the previous year allows their damaged neighbours to recover prior to the next damage event, thereby improving overall plant performance.

## 5. Conclusions

Minor and local insect damage may reduce leaf FA of the affected plant shoot in the year following the damage, in contrast to severe defoliation that frequently increases plant FA. Thus, damage by insects contributes to within-plant variation in leaf asymmetry, and may influence the distribution of herbivory in the following year.

## Figures and Tables

**Table 1:** Sources of variation in leaf fluctuating asymmetry (FA) and in percentage of leaf area damaged by insects in experimental and control shoots during the previous year.

Effect Type	Source of Variation	FA (2009)		Leaf Damage (2008)	
		Test Statistics	*p*	Test Statistics	*p*
Fixed	Treatment ^1^	*F*_1,61_ = 3.40	0.07	*F*_1,61_ = 117.4	<0.0001
	Insect group	*F*_5,40_ = 0.33	0.89	*F*_5,40_ = 2.24	0.06
	Treatment × Insect group	*F*_5,61_ = 0.69	0.63	*F*_5,61_ = 0.85	0.52
Random	Site	*χ*_1_ = 0.00	0.99	*χ*_1_ = 0.00	0.99
	Tree (Site)	*χ*_1_ = 0.07	0.80	*χ*_1_ = 2.86	0.09

^1^ Experimental vs. control shoots.

## Supplementary Materials

The following are available online at http://www.mdpi.com/2075-4450/9/2/56/s1, Table S1: Shoot-specific values of leaf FA and of previous-year losses of leaf area to insects.
