# Supplementary material for: Local Insect Damage Reduces Fluctuating Asymmetry in Next-year’s Leaves of Downy Birch
Source: Insects. 2018 May 11;9(2):56. doi: 10.3390/insects9020056 (PMC6023539; doi:10.3390/insects9020056)
Supplement: Supplementary file 1 [file insects-09-00056-s001.pdf]

Table S1. Shoot-specific values of leaf fluctuating asymmetry and of previous-year losses of leaf area of downy birch, *Betula pubescens*, to insects.

Site	Tree	Insect herbivore	Shoot type	Leaf number	Fluctuating asymmetry	Leaf area lost, %
Jakarla	3	*Stigmella* sp.	Control	2	0.17357	0.2
Jakarla	3	*Stigmella* sp.	Experimental	2	0.11593	4.1
Jakarla	9	Mining sawfly	Control	2	0.06015	0.6
Jakarla	9	Mining sawfly	Experimental	2	0.09512	14.6
Jakarla	9	*Stigmella* sp.	Control	2	0.10986	0.8
Jakarla	9	*Stigmella* sp.	Experimental	2	0.05825	5.8
Jakarla	11	Leafrolling larva	Control	2	0.03465	0
Jakarla	11	Leafrolling larva	Experimental	2	0.06893	47.5
Jakarla	11	*Deporaus betulae*	Control	2	0.10536	0
Jakarla	11	*Deporaus betulae*	Experimental	2	0.11206	46.7
Jakarla	11	*Deporaus betulae*	Control	2	0.10545	1.8
Jakarla	11	*Deporaus betulae*	Experimental	2	0.06073	14.8
Jakarla	11	*Deporaus betulae*	Control	2	0.06495	15.7
Jakarla	11	*Deporaus betulae*	Experimental	2	0.01490	46.9
Jakarla	13	*Eriocrania* sp.	Control	2	0.12179	3.0
Jakarla	13	*Eriocrania* sp.	Experimental	2	0.09111	8.7
Jakarla	13	*Eriocrania* sp.	Control	2	0.01380	0.7
Jakarla	13	*Eriocrania* sp.	Experimental	2	0.01583	8.8
Jakarla	15	*Deporaus betulae*	Control	2	0.11941	4.7
Jakarla	15	*Deporaus betulae*	Experimental	2	0.05890	16.3
Jakarla	17	Leafrolling larva	Control	2	0.23331	1.1
Jakarla	17	Leafrolling larva	Experimental	2	0.05857	20.0
Jakarla	17	*Parornix* sp.	Control	2	0.07417	3.8
Jakarla	17	*Parornix* sp.	Experimental	2	0.02348	7.6
Jakarla	17	*Parornix* sp.	Control	1	0.08106	3.5
Jakarla	17	*Parornix* sp.	Experimental	2	0.08842	23.9
Jakarla	17	*Stigmella* sp.	Control	2	0.22574	3.5
Jakarla	17	*Stigmella* sp.	Experimental	2	0.09932	14.3
Jakarla	17	*Deporaus betulae*	Control	2	0.00837	5.6
Jakarla	17	*Deporaus betulae*	Experimental	2	0.08382	19.1
Jakarla	18	*Eriocrania* sp.	Control	1	0.22861	1.6
Jakarla	18	*Eriocrania* sp.	Experimental	1	0.14736	15.5
Jakarla	19	Mining sawfly	Control	1	0.11452	0
Jakarla	19	Mining sawfly	Experimental	1	0.04193	17.8
Jakarla	20	*Eriocrania* sp.	Control	2	0.07550	2.9
Jakarla	20	*Eriocrania* sp.	Experimental	2	0.01509	8.7
Jakarla	20	*Stigmella* sp.	Control	2	0.06845	3.2
Jakarla	20	*Stigmella* sp.	Experimental	2	0.12037	20.5
Jakarla	20	*Deporaus betulae*	Control	2	0.04218	3.1

Jakarla	20	*Deporaus betulae*	Experimental	2	0.01527	8.2
Jakarla	22	*Deporaus betulae*	Control	2	0.09380	0
Jakarla	22	*Deporaus betulae*	Experimental	2	0.08146	46.7
Jakarla	23	*Parornix* sp.	Control	2	0.03578	2.5
Jakarla	23	*Parornix* sp.	Experimental	2	0.07844	6.6
Jakarla	23	*Deporaus betulae*	Control	1	0.18963	2.0
Jakarla	23	*Deporaus betulae*	Experimental	2	0.04825	20.3
Jakarla	23	*Deporaus betulae*	Control	2	0.13275	2.2
Jakarla	23	*Deporaus betulae*	Experimental	2	0.15447	16.4
Jakarla	23	*Deporaus betulae*	Control	2	0.16508	3.5
Jakarla	23	*Deporaus betulae*	Experimental	2	0.04369	14.7
Jakarla	25	*Parornix* sp.	Control	2	0.07977	0
Jakarla	25	*Parornix* sp.	Experimental	2	0.09771	1.3
Jakarla	25	*Parornix* sp.	Control	2	0.05017	1.9
Jakarla	25	*Parornix* sp.	Experimental	2	0.03591	8.1
Jakarla	25	*Stigmella* sp.	Control	2	0.20914	10.3
Jakarla	25	*Stigmella* sp.	Experimental	2	0.10582	31.6
Jakarla	28	*Stigmella* sp.	Control	2	0.05418	0
Jakarla	28	*Stigmella* sp.	Experimental	2	0.01714	5.3
Jakarla	28	*Stigmella* sp.	Control	2	0.07652	0
Jakarla	28	*Stigmella* sp.	Experimental	2	0.01945	3.0
Jakarla	29	*Stigmella* sp.	Control	2	0.05108	0
Jakarla	29	*Stigmella* sp.	Experimental	2	0.04366	4.0
Jakarla	30	*Stigmella* sp.	Control	2	0.02887	0
Jakarla	30	*Stigmella* sp.	Experimental	2	0.15759	5.5
Jakarla	32	Leafrolling larva	Control	2	0.10738	0
Jakarla	32	Leafrolling larva	Experimental	2	0.08154	7.0
Jakarla	32	*Stigmella* sp.	Control	2	0.13932	0
Jakarla	32	*Stigmella* sp.	Experimental	2	0.05923	27.5
Jakarla	34	Mining sawfly	Control	2	0.05974	3.2
Jakarla	34	Mining sawfly	Experimental	2	0.02881	10.9
Jakarla	37	*Parornix* sp.	Control	2	0.07646	0
Jakarla	37	*Parornix* sp.	Experimental	2	0.03806	31.9
Raisio	4	Mining sawfly	Control	1	0.01730	11.7
Raisio	4	Mining sawfly	Experimental	1	0.04366	44.8
Raisio	5	Mining sawfly	Control	1	0.10617	0
Raisio	5	Mining sawfly	Experimental	2	0.09571	14.5
Raisio	6	Leafrolling larva	Control	1	0.02168	3.3
Raisio	6	Leafrolling larva	Experimental	1	0.11069	27.1
Raisio	8	*Deporaus betulae*	Control	2	0.14344	1.5
Raisio	8	*Deporaus betulae*	Experimental	2	0.03055	30.4
Raisio	8	*Deporaus betulae*	Control	2	0.27675	1.3
Raisio	8	*Deporaus betulae*	Experimental	2	0.03354	30.3

Raisio	14	*Parornix* sp.	Control	2	0.11571	1.1
Raisio	14	*Parornix* sp.	Experimental	2	0.08450	5.4
Raisio	14	*Parornix* sp.	Control	2	0.09021	0
Raisio	14	*Parornix* sp.	Experimental	2	0.07265	3.9
Raisio	16	*Parornix* sp.	Control	2	0.07133	0
Raisio	16	*Parornix* sp.	Experimental	2	0.15563	5.5
Raisio	16	*Parornix* sp.	Control	1	0.03915	0.4
Raisio	16	*Parornix* sp.	Experimental	1	0.03663	13.6
Raisio	16	*Stigmella* sp.	Control	1	0.03239	0
Raisio	16	*Stigmella* sp.	Experimental	2	0.09807	1.0
Raisio	16	*Stigmella* sp.	Control	2	0.19149	0.4
Raisio	16	*Stigmella* sp.	Experimental	2	0.05109	3.9
Raisio	16	*Stigmella* sp.	Control	2	0.04122	0
Raisio	16	*Stigmella* sp.	Experimental	2	0.04566	3.5
Raisio	18	*Stigmella* sp.	Control	2	0.08553	0.6
Raisio	18	*Stigmella* sp.	Experimental	2	0.04317	18.6
Raisio	19	*Stigmella* sp.	Control	1	0.02776	0
Raisio	19	*Stigmella* sp.	Experimental	2	0.06783	2.6
